# A Novel Computational Method of Processing Map for Ti-6Al-4V Alloy and Corresponding Microstructure Study

**DOI:** 10.3390/ma11091599

**Published:** 2018-09-03

**Authors:** Ming Hu, Limin Dong, Zhiqiang Zhang, Xiaofei Lei, Rui Yang, Yuhui Sha

**Affiliations:** 1 Key Laboratory for Anisotropy and Texture of Materials (Ministry of Education), Northeastern University, Shenyang 110819, China; mhu13s@imr.ac.cn (M.H.); yhsha@mail.neu.edu.cn (Y.S.); 2 Institute of Metal Research, Chinese Academy of Sciences, Shenyang 110016, China; zqzhang@imr.ac.cn (Z.Z.); xflei@imr.ac.cn (X.L.); ryang@imr.ac.cn (R.Y.)

**Keywords:** Ti-6Al-4V alloy, hot deformation, processing map, microstructure

## Abstract

The Arrhenius-type constitutive equation is mostly used to describe flow behaviors of material. However, no processing map has been constructed directly according to it. In this study, a novel computational method was applied for establishing the processing map for Ti-6Al-4V alloy in the temperature and strain rate range of 800–1050 °C and 0.001–10 s^−1^, respectively. The processing map can be divided into four domains according to its graphic features. Among the four domains, the optimal domain is in the temperature and strain rate range of 850–925 °C and 0.001–0.1 s^−1^, where peak efficiency *η* is 0.54 and the main microstructural evolution is DRX (dynamic recrystallization). When the alloy is processed in the α + β phase field, the temperature and strain rate range of 800–850 °C and 3–10 s^−1^ should be avoided, where instability parameter *ξ* is negative and the microstructural feature is flow localization. When the alloy is processed in the β phase field, DRV (dynamic recovery) and slight DRX of β phase is the main microstructural characteristics in the range of 1000–1050 °C and 0.001–0.02 s^−1^. However, flow localization of β phase is the main microstructural feature in the range of 1000–1050 °C and 1–10 s^−1^, which should be avoided.

## 1. Introduction

The processing map proposed by Prasad et al. [1] is widely used for optimizing processing parameters and controlling microstructure in thermomechanical processing (TMP) [2,3]. The construction of processing map is based on the dynamic materials model (DMM) which considers workpiece to be a dissipater of power [4,5]. In DMM, the total power *P* is clarified into two parts: *G* and *J*. The *G* content represents the power which is converted into temperature rise. And the *J* co-content represents the power dissipated through the microstructure change, such as phase transformation, recovery, recrystallization, cavity formation and so forth [6]. The constitutive relationship decides the relative values of power dissipation through the temperature rise and microstructure change [7]. The computing method for processing map proposed by Prasad et al. [1] was derived by assuming that the constitutive relationship between flow stress σ and strain rate $\dot{\varepsilon }$ obeys the power law equation. However, in most literature [2,8,9,10], the method was directly used for constructing processing maps without considering whether the specific constitutive relationships satisfy the power law equation. The computation of processing maps may become erroneous if specific constitutive relationships are ignored. Therefore, Murty [11] proposed a computing method for processing a map suitable for any metals and alloys, as it does not include the constitutive relationships between σ and $\dot{\varepsilon }$. This method has been successfully applied in Zr-2.5Nb-0.5Cu alloy [11]. Although there are many advantages of this method, the application is limited due to the difficulty in integral calculations. Because of the limitation or inconvenience of the above methods, a more suitable method should be explored. As known, the Arrhenius-type constitutive equation is most widely used to describe the relationship between the $\dot{\varepsilon }$, σ and temperature [12]. However, no processing map has been constructed based on this model. Therefore, in this paper, a novel computing method for processing map was introduced based on the Arrhenius-type constitutive equation.

The two-phase α/β titanium alloy Ti-6Al-4V has been widely used in the aerospace industry due to its high strength-to-weight ratio, good corrosion resistance and excellent mechanical properties. However, there are many difficulties in controlling its microstructure and mechanical properties due to the narrow processing time and temperature window [13]. As mentioned above, processing map is an excellent tool for processing parameters optimization and microstructure control. Although the processing maps of Ti-6Al-4V alloy have been widely studied [14,15,16], their applied computing methods are based on the traditional power law equation.

In this study, isothermal hot compression tests were conducted in the temperature range of 800–1050 °C and the strain rate range of 0.001–10 s^−1^. The computing method of processing maps based on the traditional power law equation and the Arrhenius-type constitutive equation were compared. The results indicate that the novel processing map is more effective. According to the present processing map, the stable domains and unstable domains were clarified and were validated by the microstructural observations. Eventually, optimized processing parameters for hot deformation of Ti-6Al-4V alloy was suggested.

## 2. Materials and Methods

The material used in this study was a hot-rolled and annealed Ti-6Al-4V alloy bar with a nominal chemical composition of Ti-6.2Al-4.6V. The β-transus temperature was measured at about 995 °C using the metallographic method. Cylindrical specimens with 8 mm diameter and 12 mm height were machined from the annealed bar. The deformation temperature ranged from 800 °C to 1050 °C at an interval of 50 °C. The stain rates were 0.001 s^−1^, 0.01 s^−1^, 0.1 s^−1^, 1 s^−1^ and 10 s^−1^ respectively. Specimens were heated to the deformation temperatures at a rate of 5 °C/s and held for 5 min using a Gleeble-3500 Thermal/Mechanical Simulator (Dynamic Systems Inc., New York, NY, USA). Subsequently, specimens were isothermally compressed up to total true strain of 0.9 and air cooled to room temperature. A thin layer of graphite was used to minimize the friction between the specimen and the anvils. Partial compression results (stain rates 0.001 s^−1^, 0.01 s^−1^, 0.1 s^−1^ and 1 s^−1^) are from previous study [17], in which only constitutive models were established. A processing map was further constructed based on the Arrhenius-type constitutive equation [17] in this study. For comparison, a processing map was also constructed based on the traditional power law equation.

Microstructural observations of the compressed specimens were made on the sectional plane parallel to the compression axis in the central regions. For scanning electron microscopy (SEM, Zeiss Merlin Compact, Jena, Thuringgia, Germany) observations, specimen surfaces were prepared by metallographical polishing and followed by chemical etching in a solution consisting of 5% HF + 5% HNO_3_ + 85% H_2_O for 10 s. Transmission electron microscopy (TEM, Tecnai G^2^ 20, Field Electron and Ion., Hillsboro, OR, USA) samples were prepared by mechanical grinding to about 40 μm followed by twin-jet electropolishing (Tenupol-5, Struers, Copenhagen, Denmark) using the solution of 6% perchloric acid, 59% methyl alcohol and 30% mutual. Electron backscatter diffraction (EBSD, Oxford Instruments, Abingdon, UK) samples were made, first with metallographically polishing and finally by being electro-polished in the same solution with TEM. The EBSD data was analyzed by the tango program using the HKL-Channel 5.

## 3. Results and Discussion

### 3.1. Flow Behavior

**Figure 1:**
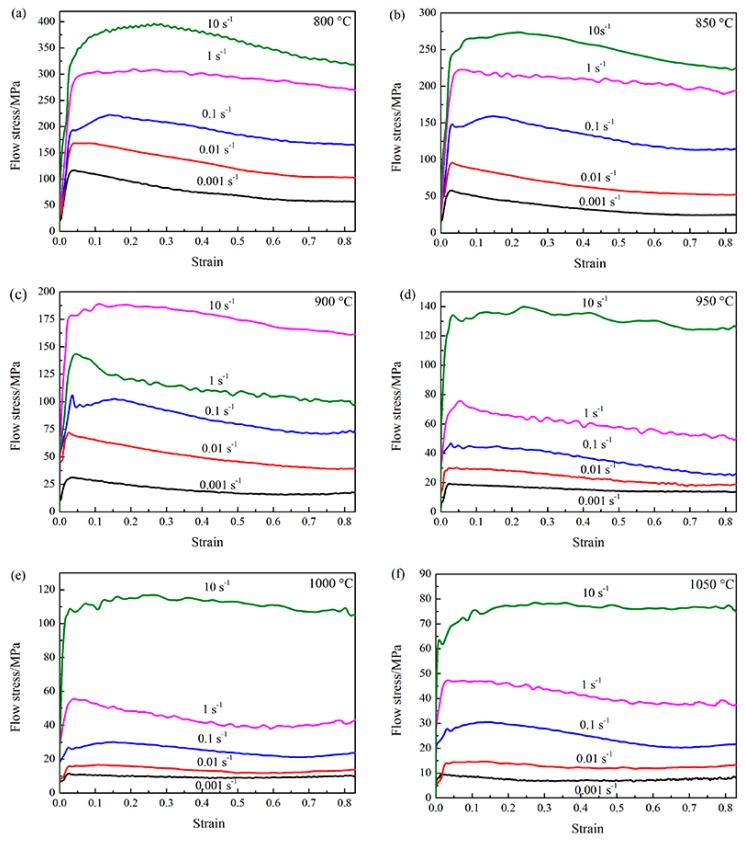
Flow stress curves of isothermally compressed Ti-6Al-4V alloy at different deformation temperatures: (**a**) 800 °C; (**b**) 850 °C; (**c**) 900 °C; (**d**) 950 °C; (**e**) 1000 °C; (**f**) 1050 °C.

The flow curves in Figure 1 were smoothed and then corrected by considering the effect of friction and temperature rise. The flow curves show flow stress are significantly influenced by strain rates and temperatures. Flow stress decreases with the increasing of the deformation temperature at a constant deformation strain rate. In this case, the increasing of temperature increases the kinetic energy of atoms and thus decrease the critical shear stress for slip. The volume fraction of softer β phase increases with the increasing of the deformation temperature. This also contributes to the decreasing of the flow stress. On the other hand, the flow stress increases with the increasing of deformation strain rate at a constant deformation temperature. The main reason is that the level of dynamic softening is lower in high strain rates.

The flow curves show different shapes in Figure 1. The different shapes of stress-strain curves are the results of competition between work hardening and dynamic softening. The mechanism of dynamic softening generally includes dynamic recrystallization (DRX), dynamic recovery (DRV) or flow instability [18,19,20]. For all flow curves, the dislocation density grows rapidly and the flow stress increases sharply to a peak stress at a small strain at the initial deformation stage [21]. Beyond the peak stress, the curves exhibit steady-state type (e.g., 1050 °C, 0.001 s^−1^) or flow softening (e.g., 800 °C, 0.001 s^−1^) when dynamic softening balances or exceeds the work hardening. In addition, the sudden drop of flow stress (e.g., 900 °C, 0.1 s^−1^) beyond the peak stress called the discontinuous yielding behavior was observed. The discontinuous yielding behavior has been found in many titanium alloys, such as Ti55 [22], Ti60 [20], Ti-6.5Al-3.5Mo-0.25Si [23] and Ti-10V-4.5Fe-1.5Al [24]. The main reason for this phenomenon is the abrupt formation of large quantities of new mobile dislocations originating from the grain boundary sources [25].

### 3.2. Construction of Processing Map

In the DMM, the *G* content, *J* co-content and total power *P* can be expressed in Equation (1) [1]:

(1) $$\begin{array}{l} G=\int_{0}^{\dot{\varepsilon }}\sigma d\dot{\varepsilon }\\ J=\int_{0}^{\sigma }\dot{\varepsilon }d\sigma \\ P=\sigma \dot{\varepsilon }=G+J \end{array}$$

where *σ* is the flow stress (MPa), $\dot{\varepsilon }$ is the strain rate (s^−1^). When workpiece is an ideal linear dissipater, the *J* reaches its maximum value (i.e., $J=J_{max}=\sigma \dot{\varepsilon }/2$). By comparing the non-linear power dissipation capacity of workpiece during deformation, the efficiency of power dissipation *η* is defined as:

(2) $$\eta =\frac{J}{J_{\max}}$$

The efficiency of power dissipation *η* can be used to represent the power dissipated through microstructure change. The variations of *η* values with strain rate and temperature constitute the power dissipation map at a given strain. The domains with high *η* values are considered to be safe domains and microstructure mechanisms are believed to be DRX, DRV, spheroidization or superplasticity (SP) [26]. However, it is inadequate to identify the safe domains and unsafe domains only according to *η* values [1]. Therefore, a continuum instability criterion proposed by Ziegler [11] is applied to large plastic deformation to identify unsafe domains, as given in Equation (3). D is the dissipative function. Prasad used *J* to replace *D* in Equation (3), then derived Equation (4) [11].

(3) $$\frac{dD}{d\dot{\varepsilon }}<\frac{D}{\dot{\varepsilon }}$$

(4) $$\xi (\dot{\varepsilon })=\frac{\partial \ln J}{\partial \ln\dot{\varepsilon }}-1\leq 0$$

The variations of *ξ* values with strain rate and temperature constitute the instability map which depicts instability regions. In the negative *ξ* values regions, microstructure mechanisms are believed to be adiabatic shear band, flow localization, dynamic strain aging or twinning. Eventually, the processing map is established by superimposing the instability map over the power dissipation map.

In this study, the true strain of 0.8 is close to the true strain of final compression state. Therefore, this strain was used to construct the processing map. In this section, both methods of the traditional power law equation and Arrhenius-type constitutive equation were carried out to compute the processing maps. Then, the effectiveness of the two methods was compared.

#### 3.2.1. Processing Map Based on the Traditional Power Law Constitutive Equation

In this section, the processing map was directly constructed by using the method proposed by Prasad et al. [1]. But the effectiveness of power law constitutive equation for Ti-6Al-4V alloy was not verified.

The power law constitutive equation was shown in Equation (5).

(5) $$\sigma =K{\dot{\varepsilon }}^{m}$$

Then, the values of *η* and *ξ* can be easily derived by combining Equations (1)–(4) and Equation (5), as expressed in Equation (6) and Equation (7).

(6) $$\eta =\frac{J}{J_{\max}}=\frac{\int_{0}^{\sigma }\dot{\varepsilon }d\sigma }{\sigma \dot{\varepsilon }/2}=\frac{m\sigma \dot{\varepsilon }/(m+1)}{\sigma \dot{\varepsilon }/2}=\frac{2m}{m+1}$$

(7) $$\xi (\dot{\varepsilon })=\frac{\partial \ln[m/(m+1)]}{\partial \ln\dot{\varepsilon }}+m\leq 0$$

For calculating the values of *η* and *ξ,* the value of strain rate sensitivity exponent *m* should be acquired first. The value of *m* can be obtained using Equation (8) by taking natural logarithm of both sides of Equation (5).

(8) $$m={\left(\frac{\partial \ln\sigma \;}{\partial \ln\dot{\varepsilon }}\right)}_{\varepsilon ,T}$$

For a given strain and temperature, the plot of lnσ versus ln $\dot{\varepsilon }$ can be fitted by a cubic spline. The algebraic expression can be presented by a third order polynomial, as shown in Equation (9).

(9) $$\ln\sigma =k_{1}+k_{2}\ln\dot{\varepsilon }+k_{3}{\left(\ln\dot{\varepsilon }\right)}^{2}+k_{4}{\left(\ln\dot{\varepsilon }\right)}^{3}$$

Substituting Equation (9) into Equation (8) yields Equation (10):

(10) $$m=k_{2}+2k_{3}(\ln\dot{\varepsilon })+3k_{4}{\left(\ln\dot{\varepsilon }\right)}^{2}$$

**Figure 2:**
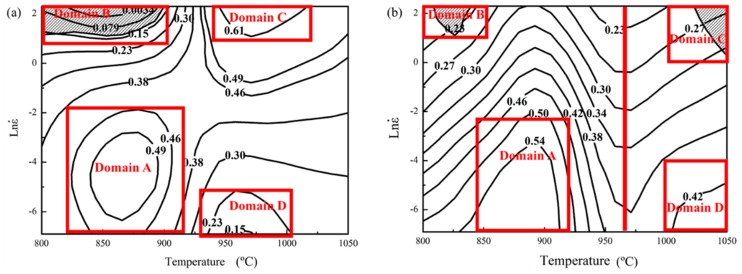
Processing maps for Ti-6Al-4V alloy derived based on (**a**) power law constitutive equation and (**b**) Arrhenius-type constitutive equation.

Then, the values of *η* and *ξ* can be easily derived by combining Equations (6), (7) and (10). Eventually, the processing map was established, as shown in Figure 2a. The contour numbers represent the values of *η* and the shaded regime is the unstable domains with negative *ξ* values. In Figure 2a, the processing map is divided into four domains. Domain A approximately lies in the temperature and strain rate range of 825–910 °C and 0.001–0.1 s^−1^. The power dissipation efficiency *η* is about 0.49 in the domain. Domain B approximately locates in the range of 800–900 °C and 3–10 s^−1^. The values of *ξ* is negative in the domain. Domain C is approximately in the range of 930–1020 °C and 3–10 s^−1^. The peak efficiency *η* is 0.61 in the domain. Domain D approximately spreads over the range of 930–1000 °C and 0.001–0.01 s^−1^. The power dissipation efficiency *η* is about 0.2 in the domain.

#### 3.2.2. The Novel Processing Map Based on the Arrhenius-Type Constitutive Equation

In the previous study [17], the Arrhenius-type constitutive equation has been verified to be suitable to describe the flow behavior of Ti-6Al-4V alloy and the details of the Arrhenius-type constitutive equation were presented. In this section, a novel computing method of processing map based on the Arrhenius-type constitutive equation is proposed. The method is shown as follows.

The Arrhenius-type constitutive equation is shown in Equation (11).

(11) $$\begin{array}{l} \dot{\varepsilon }=\mathrm{AF}(\sigma ),\exp\left(-\frac{Q}{RT}\right)\\ F\left(\sigma \right)=\{\begin{array}{ll} \sigma^{n\text{′}} & \alpha \sigma <0.8\\ \exp\left(\beta \sigma \right) & \alpha \sigma >1.2\\ {\left[\sinh\left(\alpha \sigma \right)\right]}^{n} & for\;all\;\sigma \end{array}, \end{array}$$

where *Q* is the activation energy (kJ·mol^−1^), *R* is the universal gas constant (8.3145 J·mol^−1^·K^−1^), *T* is the absolute temperature (K), A, n′, β, α and n are the material constants.

From Equations (1) and (11), the dissipater power co-content *J* can be represented as:

(12) $$J=\int_{0}^{\sigma }\dot{\varepsilon }d\sigma =\int_{0}^{\sigma }A[\sin h(\alpha \sigma {)]}^{n}\exp(-\frac{Q}{RT})d\sigma ,$$

The value of *η* was calculated as Equation (13):

(13) $$\eta =\frac{J}{J_{\max}}=\frac{\int_{0}^{\sigma }A[\sin h(\alpha \sigma {)]}^{n}\exp(-\frac{Q}{RT})d\sigma }{\sigma \dot{\varepsilon }/2}$$

The value of *η* can be achieved by substituting the Arrhenius-type constitutive equation into Equation (13). The integral of the Arrhenius-type constitutive equation in Equation (13) can be easily calculated with MATLAB R2016a using function int.

In Equation (4), the value of *ξ* is difficult to be calculated directly. The computational method proposed by Murty [11] was employed to calculate the values of *ξ*, as expressed in Equation (14).

(14) $$\xi =\frac{mP}{J}-1=\frac{2m}{\eta }-1<0$$

The values of strain rate sensitivity exponent *m* can be calculated as Equation (15):

(15) $${\left(\frac{\partial J}{\partial G}\right)}_{\varepsilon ,T}=\frac{\dot{\varepsilon }}{\sigma }\frac{d\sigma }{d\dot{\varepsilon }}=m$$

In order to obtain *m* in Equation (15), the value of dσ/d $\dot{\varepsilon }$ should be calculated. The σ can be represented as Equation (16) by parameters of α, Z, A and n. The values of dσ/d $\dot{\varepsilon }$ can be calculated using MATLAB (The program was presented in the Appendix A).

(16) $$\begin{array}{l} Z=\dot{\varepsilon }\exp\left(\frac{Q}{RT}\right)\\ \sigma =\frac{1}{\alpha }\ln\left\{{\left(\frac{Z}{A}\right)}^{1/n}+{\left[{\left(\frac{Z}{A}\right)}^{2/n}+1\right]}^{1/2}\right\} \end{array}$$

Then, the value of *ξ* can be calculated by substituting *m* and *η* into Equation (14). Eventually, the processing map was established by superimposing the instability map over the power dissipation map, as shown in Figure 2b. The processing map is divided into four domains. Domain A lies approximately in the temperature and strain rate range of 850–925 °C and 0.001–0.1 s^−1^. The peak efficiency *η* is 0.54 in the domain. The domain is considered to be safe domain. Microstructure mechanisms such as DRX and DRV may occur in this domain. Domain B locates approximately in the range of 800–850 °C and 3–10 s^−1^. The values of *ξ* are negative in the domain. Flow instability mechanisms such as the adiabatic shear band and flow localization may occur in this domain. Domain C lies approximately in the range of 1000–1050 °C and 1–10 s^−1^. Domain D locates approximately in the range of 1000–1050 °C and 0.001–0.02 s^−1^. The power dissipation efficiency *η* is about 0.42 in the domain. In addition, the iso-efficiency contours in Figure 2b show a distinct change in their curvature close to 970 °C (the red line). This feature is considered to be related to the dramatic variations of the α/β phase proportion. This phenomenon was frequently observed in all materials which show phase transformation [16].

#### 3.2.3. Effectiveness Comparison of Processing Maps

Although both processing maps can be divided into four domains, there are many differences in the values and scopes of *η* and *ξ.* The maximum power dissipation efficiency 0.54 occurs in Domain A in Figure 2b. However, the maximum power dissipation efficiency 0.61 occurs in Domain C in Figure 2a. It is surprising that maximum power dissipation efficiency occurs at the high strain rate in Figure 2a. It can be seen that Domain C in Figure 2b was flow instability region but this Domain C is safe domain in Figure 2a. Generally, power dissipation efficiency is smaller at high strain rate where adiabatic shear band or flow localization may occur. The value of *η* is 0.42 in Domain D in Figure 2b. However, the value of *η* is smaller in Domain D in Figure 2a. Generally, power dissipation efficiency is higher at low strain rate where DRX or DRV may occur. Thus, the results in Figure 2a are incorrect. The reasons for the incorrect results may be related to the power law constitutive equation for Ti-6Al-4V alloy in this study. Therefore, the results of Figure 2b agree with this argument and fit engineering reality much better.

### 3.3. Identification of Microstructure in Different Domains of the Novel Processing Map

It is commonly acknowledged that the processing map alone is not sufficient to determine the optimum processing parameters and it is needed to be confirmed through microstructural characterization [2]. Thus, microstructural characterizations corresponding to different domains in Figure 2b were studied in the following section. The SEM, EBSD and TEM results were used to show the microstructure of different domains in the processing map.

#### 3.3.1. Microstructural Characterizations for As-Received Specimen

**Figure 3:**
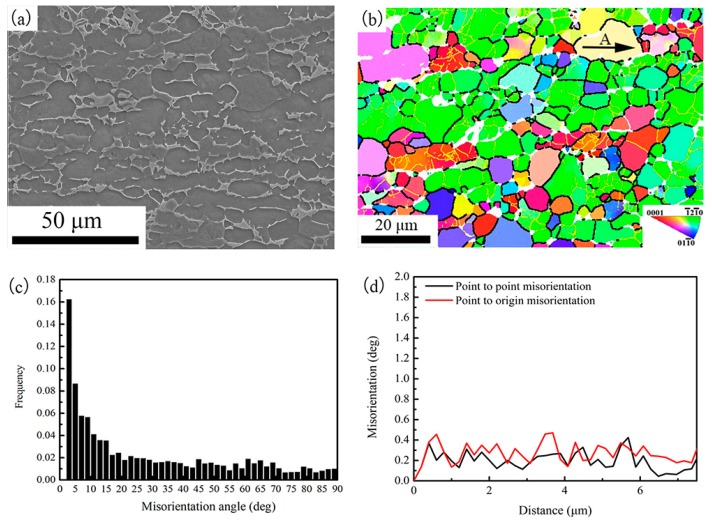
(**a**) SEM image; (**b**) IPF map; (**c**) Misorientation distribution and (**d**) Misorientation development along arrow A in (**b**) for the as-received specimen.

The SEM image and IPF (inverse pole figure) map of the as-received specimen are shown in Figure 3a,b, respectively. The grains are elongated in rolling direction. The average grain size is 15 μm in length direction and 6 μm in transverse direction. Figure 3b also shows the low-angle boundaries (LABS, depicted by yellow line) and high-angle boundaries (HABS, depicted by black line) of the as-received specimen. A large number of LABS were observed. Figure 3c shows the misorientation distribution. It also indicates most boundaries are LABS. The misorientation profile inside the grain along the arrow A in Figure 3b was evaluated and the results are shown in Figure 3d. Fluctuations in both local misorientation (point-to-point) and cumulative misorientation (point-to-origin) were observed and the maximum misorientation is less than 0.6°. It indicates that the grains are strain-free recrystallized structure. Thus, the crystal structure of the as-received specimen consists of the recovered and recrystallized structures.

#### 3.3.2. Microstructural Evolution of Domain A and B in the α + β Field

**Figure 4:**
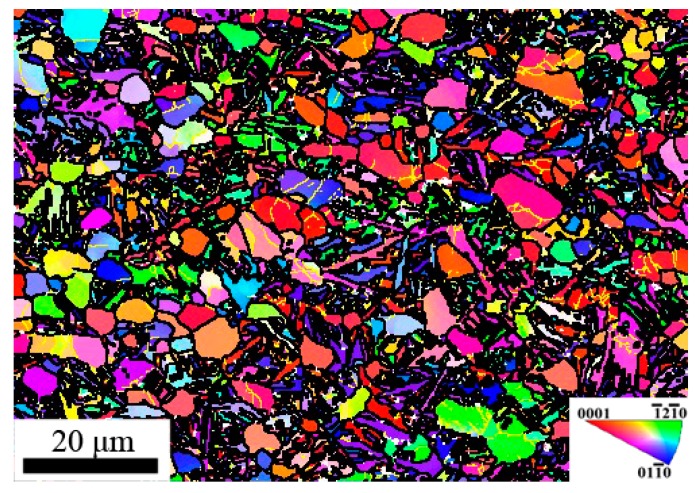
IPF map of specimen compressed at 900 °C and 0.01 s^−1.^

The IPF map of compressed specimen corresponding to the Domain A is represented in Figure 4. Substantial fine and equiaxed DRX grains were found in this domain, which is consistent with the higher efficiency of power dissipation 0.54. Meanwhile, significant flow softening was observed in flow curves of Figure 1c since the dynamic softening caused by DRX exceeds work hardening. Thus, DRX is the main microstructural mechanism in this domain. So, this domain was considered to be the optimal hot working window.

**Figure 5:**
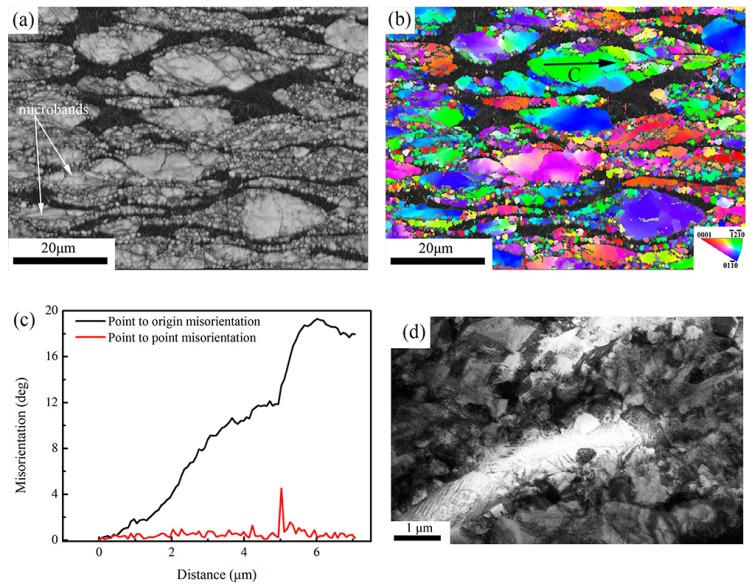
(**a**) IQ map; (**b**) IPF map; (**c**) Misorientation development along arrow A in (**b**,**d**) transmission electron microscopy (TEM) image for specimen compressed at 800 °C and 10 s^−1^.

Figure 5a,b,d show the IQ (image quality) map, IPF map and TEM graph of specimen deformed in the domain B. It can be observed that some parts of the regions are black in Figure 5a,b, because these regions have undergone severe lattice distortion and this cannot be resolved by EBSD. Meanwhile, many fine grains were observed due to boundary splitting associated with instability between interphase α/β boundaries and intraphase α/α boundaries in Figure 5a,b. In Figure 5a, a large amount of parallel deformation directions, oriented 45° to compression axis microbands, were observed within grains. It indicates that the localized shear deformation across α phase is the main reason for the formation of intraphase α/α boundaries. The misorientation profile along the arrows of D in Figure 5b, is shown in Figure 5c. It can be observed that maximum cumulative misorientation within the deformed grains exceeds 19°, which is much larger than that of the initial state. Many fine grains were also observed in the TEM graph of Figure 5d. Thus, flow localization is the main instability mechanism in this domain.

#### 3.3.3. Microstructural Evolution of Domain C and D in the β Field

**Figure 6:**
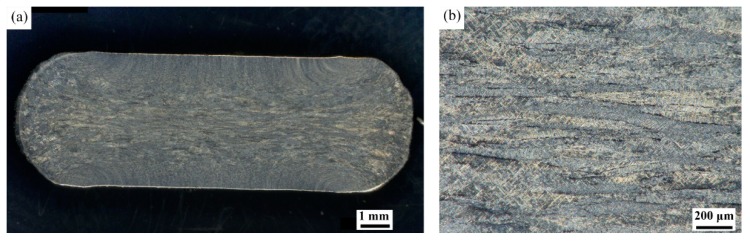
(**a**) Macrostructure image and (**b**) central region photo of Figure 6a on higher magnification of specimen compressed at 1050 °C and 10 s^−1^.

Figure 6a represents the macrostructure of sample deformed in the domain C. Figure 6b is the higher magnification of the central region in Figure 6a. As it can be seen, the grains are elongated in flow direction. No DRX is observed in the central region. Meanwhile, flow curve in Figure 1f is steady-state. Flow softening is balanced with the work hardening. At high strain rate, flow instability is the possible mechanism of softening. The negative *ξ* values also indicate flow instability in domain C. No micro-cracking is found in this domain. Thus, β phase flow localization is the main mechanism of softening in this domain.

**Figure 7:**
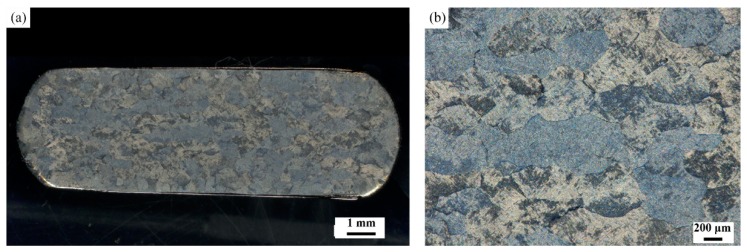
(**a**) Macrostructure image and (**b**) central region photo of Figure 7a on higher magnification of specimen compressed at 1050 °C and 0.001 s^−1^.

Figure 7a shows the macrostructure of sample deformed in the Domain D. Figure 7b is the higher magnification of the central region in Figure 7a. Many coarse grains mixed by a few fine grains were observed. The boundaries of deformed grains are curved. Prasad and Seshacharyulu [27] have found that the starting microstructure, consisting of equiaxed α + β structures when formed in the β phase field, does not contain a stable subgrain structure to cause large-grained SP. Meanwhile, the power dissipation efficiency 0.42 is higher in the domain. Thus, DRV and a few DRX of β phase is the main microstructural characteristics in this domain.

## 4. Conclusions

In this study, the hot deformation characteristics of Ti-6Al-4V alloy were investigated using isothermal compression tests in the temperature range of 800–1050 °C and strain rate range of 0.001–10 s^−1^. Flow behaviors were analyzed. The processing maps based on the traditional power law equation and Arrhenius-type constitutive equation were constructed and compared. Microstructural characteristics in different domains were studied. The results of microstructure characterization agree well with the results of the novel processing map. The following conclusions are drawn from this study.

(1) A novel computing method of processing map was constructed according to the Arrhenius-type constitutive equation. The results of the novel processing map fit engineering reality better than the traditional processing map.

(2) The novel processing map was divided into four domains according to its graphic features. The peak efficiency with a maximum value 0.54 lies in the temperature and strain rate range of 850–925 °C and 0.001–0.1 s^−1^.

(3) In the α + β phase field, DRX is the main microstructural characteristics in the temperature and strain rate range of 850–925 °C and 0.001–0.1 s^−1^. This domain is considered to be the optimal hot working window. However, flow localization occurs in the temperature and strain rate range of 800–850 °C and 3–10 s^−1^.

(4) In the β phase field, DRV and slight DRX of β phase are the main microstructural feature in the temperature and strain rate range of 1000–1050 °C and 0.001–0.02 s^−1^. Flow localization of β phase is the main microstructural characteristics in the temperature and strain rate range of 1000–1050 °C and 1–10 s^−1^.

## Appendix A

syms x a w b q t %, x is strain rate, a, q and t are parameters in Equation (10). w and b are parameters for calculate *A* and *n* Equation (16). w is equal to A and b is equal to 1/n.

z = x*exp(q/(8.314*t));

y = a*log((z/(w)).^b + ((z/(w)).^(2*b) + 1).^0.5);

df1 = diff(y,x);

% Calculation of dσ/d $\dot{\varepsilon }$.
